# Activation of human B cells by the agonist CD40 antibody CP-870,893 and augmentation with simultaneous toll-like receptor 9 stimulation

**DOI:** 10.1186/1479-5876-7-93

**Published:** 2009-11-11

**Authors:** Erica L Carpenter, Rosemarie Mick, Jens Rüter, Robert H Vonderheide

**Affiliations:** 1 Abramson Family Cancer Research Institute, University of Pennsylvania School of Medicine, Philadelphia, PA 19104, USA; 2 Abramson Cancer Center, University of Pennsylvania School of Medicine, Philadelphia, PA 19104, USA; 3 Division of Hematology-Oncology, Department of Medicine, University of Pennsylvania School of Medicine, Philadelphia, PA 19104, USA; 4 Department of Biostatistics and Epidemiology, University of Pennsylvania School of Medicine; Philadelphia, PA 19104, USA

## Abstract

**Background:**

CD40 activation of antigen presenting cells (APC) such as dendritic cells (DC) and B cells plays an important role in immunological licensing of T cell immunity. Agonist CD40 antibodies have been previously shown in murine models to activate APC and enhance tumor immunity; in humans, CD40-activated DC and B cells induce tumor-specific T cells in vitro. Although clinical translation of these findings for patients with cancer has been previously limited due to the lack of a suitable and available drug, promising clinical results are now emerging from phase I studies of the agonist CD40 monoclonal antibody CP-870,893. The most prominent pharmacodynamic effect of CP-870,893 infusion is peripheral B cell modulation, but direct evidence of CP-870,893-mediated B cell activation and the potential impact on T cell reactivity has not been reported, despite increasing evidence that B cells, like DC, regulate cellular immunity.

**Methods:**

Purified total CD19+ B cells, CD19+ CD27+ memory, or CD19+ CD27^neg ^subsets from peripheral blood were stimulated in vitro with CP-870,893, in the presence or absence of the toll like receptor 9 (TLR9) ligand CpG oligodeoxynucleotide (ODN). B cell surface molecule expression and cytokine secretion were evaluated using flow cytometry. Activated B cells were used as stimulators in mixed lymphocyte reactions to evaluate their ability to induce allogeneic T cell responses.

**Results:**

Incubation with CP-870,893 activated B cells, including both memory and naïve B cells, as demonstrated by upregulation of CD86, CD70, CD40, and MHC class I and II. CP-870,893-activated B cells induced T cell proliferation and T cell secretion of effector cytokines including IFN-gamma and IL-2. These effects were increased by TLR9 co-stimulation via a CpG ODN identical in sequence to a well-studied clinical grade reagent.

**Conclusion:**

The CD40 mAb CP-870,893 activates both memory and naïve B cells and triggers their T cell stimulatory capacity. Simultaneous TLR9 ligation augments the effect of CP-870,893 alone. These results provide further rationale for combining CD40 and TLR9 activation using available clinical reagents in strategies of novel tumor immunotherapy.

## Background

The activation status of host antigen presenting cells (APC) critically determines the quality and effectiveness of T cell immune responses. Resting APC may drive T cell tolerance and anergy, but fully activated APC - classically termed "licensed APC" - autonomously trigger effective and productive T cell responses [1]. This paradigm holds true for both dendritic cells (DC) and B cells. Among the many microenvironmental factors now appreciated to contribute to APC licensing, ligation of the cell surface molecule CD40 on the surface of both DC and B cells is fundamental, particularly for tumor immunity [2-8].

CD40 is a member of the tumor necrosis factor receptor (TNF) superfamily and is broadly expressed by immune and other normal cells [9]. CD40 itself lacks intrinsic signal-transduction activity and mediates its effects via downstream adapter molecules that regulate gene expression. CD40-ligand (CD40L), also known as CD154, is the chief ligand for CD40 and is expressed primarily by activated T cells and platelets [10,11]. The interaction of CD40 and CD40L represents a major component of T cell help. Ligation of CD40 on DC, for example, induces increased surface expression of costimulatory and MHC molecules, production of proinflammatory cytokines, and enhanced T cell triggering [11,12]. CD40 ligation on resting B cells increases antigen-presenting function and proliferation [11,12].

In mice, agonist CD40 antibodies have been shown to mimic the signal of CD40L and substitute for the function of CD4+ helper T cells in experimental systems testing T cell-mediated immunity [2-4]. In tumor-bearing mice, agonist CD40 antibodies overcome T cell tolerance, evoke effective cytotoxic T cell responses, and enhance efficacy of anti-tumor vaccines [5-7]. Toll-like receptor (TLR) signalling can cooperate with CD40 activation in this regard; for example, co-administration of CD40 and TLR9 ligands in mice elicits a more effective anti-melanoma response than either ligand alone [13]. Despite these landmark studies, the clinical translational of CD40 activation in cancer patients has been limited, owing primarily to the lack of an appropriate and available drug.

CP-870,893 is a fully human, selective agonist CD40 mAb and has shown early clinical promise in phase I trials, particularly in patients with advanced melanoma [14]. Little direct evidence is available regarding its mechanism of action and in particular, its biological effects on patient APC. The primary clinical side effect of CP-870,893 infusion has been mild to moderate cytokine release syndrome, manifesting as transient fever, chills, and rigor within minutes to hours after the end of the CP-870,893 infusion and associated with acute elevations in serum IL-6 and TNF-alpha [14]. The primary pharmacodynamic effect has been rapid depletion of circulating CD19+ B cells and a suggestion of global B cell activation as evidenced by significant upregulation of CD86 expression on B cells after infusion [14] (JR and RHV, unpublished observations). This pharmacodynamic effect on B cells is particularly interesting in light of increasing evidence that B cells can regulate tumor cellular immunity. Recent findings in murine models demonstrate that tumor immune surveillance and immunotherapy are enhanced in the absence of B cells [15-19], potentially due to the elimination of suppressive or regulatory B cells [18,20]. B cells have been shown to be tolerogenic when deprived of signaling via CD40 [21].

Although in vitro effects of CP-870,893 on human DC have been reported [22], its effects on purified B cells have not been described. Here, we evaluated the in vitro effects of CP-870,893 on peripheral blood B cells from normal donors, including both memory and naïve B cells as defined by the presence or absence of CD27 expression. We studied the effect of CP-870,893 on B cell activation and B cell stimulation of T cells, and we analyzed the effects of co-stimulating B cells with the TLR9 agonist CpG ODN 2006.

## Materials and methods

### Human Peripheral Blood and Lymphocyte Isolation

Protocols approved by the Institutional Review Board of the Hospital at the University of Pennsylvania were used to obtain signed, informed consent from normal donors from whom peripheral blood was drawn. CD19+ B cells were isolated from peripheral blood mononuclear cells (PBMC) by MACS magnetic column and the B cell Isolation Kit II human (Miltenyi Biotec, Auburn, CA). Purity of isolated CD19+ B cells was >95% with contaminating DC always either undetectable or <0.2% of cells in the isolated B cell population as evaluated by expression of CD123 or CD11c. CD19+ CD27+ or CD19+ CD27^neg ^subsets were further purified using CD27 Microbeads (Miltenyi). Purified CD4+ T cells (>95%) were obtained using the CD4+ T cell Isolation Kit human (Miltenyi) and labeled with 5 uM CFSE (Molecular Probes, Eugene, OR) in PBS at a concentration of 10^7 ^cells/ml.

### B cell Culture and Activation

Cell culture was conducted using X-VIVO 15 media (Lonza, Allendale, NJ) supplemented with 10% heat-inactivated (56°C, 30 min) human AB serum, 2 mmol/L L-glutamine, 15 ug/ml gentamicin, and 20 mmol/L HEPES. Total CD19+ B cells, CD19+ CD27+ B cells, or CD19+ CD27^neg ^B cells were incubated in a 5% CO_2 _incubator at 37°C in 96-well round-bottom plates at a concentration of 10^5 ^cells/100 ul in the presence of either CP-870,893 (kindly provided by Pfizer, New London, CT), or type B CpG oligodeoxynucleotide (ODN) 2006 (InvivoGen, San Diego, CA), both CP-870,893 and CpG ODN 2006, or human IgG2 kappa (hIgG2) (Chemicon International, Temecula, CA) and ODN 2006 control (InvivoGen) as negative controls. After 48 hr, undiluted culture supernatant was collected for the detection of cytokines using BD Cytometric Bead Array Human Inflammatory Cytokine Kit (BD Biosciences, San Jose, CA) and cells were washed and either surface stained or used as stimulators in mixed lymphocyte reaction (MLR) experiments.

### Flow Cytometry

Cell surface molecule expression was evaluated by flow cytometry using a FACSCanto cytometer and FACSDiva software (BD Biosciences) and the following mouse anti-human mAb: CD40 (AbD Serotec, Raleigh, NC); and CD19, CD14, CD3, CD27, CD86, HLA-A, B, C, HLA-DR, CD70, CD11c, and CD123 (BD Biosciences). Non-viable cells were excluded on the basis of staining with the nucleic acid dye 7-amino-actinomycin D (BD Bioscience). The CD40 staining antibody from AbD Serotec is not blocked by CP-870,893, suggesting distinct binding sites that allow for measurement of CD40 expression with AbD Serotec anti-CD40 despite stimulation with CP-870,893. This was established by incubating human peripheral blood B cells in the presence of increasing concentrations of CP-870,893 or purified human IgG2 (from zero to 10 ug/ml), washing the cells, then labelling with either Abd Serotec anti-CD40 mAb or a second anti-CD40 from Invitrogen (Carlsbad, CA)). We found by flow cytometry that the mean fluorescence intensity of Abd Serotech anti-CD40 mAb was the same for preincubation with CP-870,893 or IgG2 at any concentration; in contrast, labelling with the Invitrogen anti-CD40 mAb was inhibited by >90% at 10 ug/ml or 1 ug/ml of CP-870,893 (half maximal inhibition at about 0.1 ug/ml) but not by human IgG2 at any concentration.

### Mixed Lymphocyte Reaction

B cells stimulated for 48 hr were irradiated (3000 rad) and replated at 10^5 ^cells/100 ul in the presence of purified, allogeneic, CFSE-labeled CD4+ T cells at the indicated B cell:T cell ratios. Culture supernatant was collected after 5 days and preserved at -80°C until analysis for the presence of cytokines using Cytometric Bead Array Th1/Th2 Cytokine Kit II (BD Biosciences). Flow cytometry was used to evaluate T cell proliferation by measuring the proportion of CD4+ 7-amino-actinomycin D^neg ^CFSE^low ^cells on day 5.

### Statistical Methods

Linear mixed effects regression was employed to assess the individual effects of CP-870,893 and CpG ODN 2006 and interaction between the two reagents on B cell surface marker expression and cytokine secretion, as well as T cell proliferation and cytokine secretion from the MLR. The mixed effects model estimates the fixed effects (e.g., CP-870,893 and CpG ODN 2006) while adjusting for the random effect due to the correlation among outcomes derived from a single donor's B cells being exposed to each of the four conditions [23]. Group specific comparisons of CP-870,893 or CpG ODN 2006 vs. negative controls were obtained directly from the mixed effects linear model using the *xtmixed *command in STATA v10.0 (StataCorp., College Station, TX). Group specific comparisons of CP-870,893 or CpG ODN 2006 vs. CP-870,893 plus CpG ODN 2006 were obtained from the STATA post-estimation command *lincom*. Outcomes were natural log transformed prior to modelling. *P *< 0.05 was considered to be statistically significant. Tests of interaction between CP-870,893 and CpG ODN 2006, specifically to test for more-than-additive effect on the natural log scale, were one-sided. All other tests were two-sided.

## Results

### Optimal in vitro concentration of CP-870,893 and comparison to concentrations achieved in cancer patients at the CP-870,893 maximum tolerated dose

**Figure 1:**
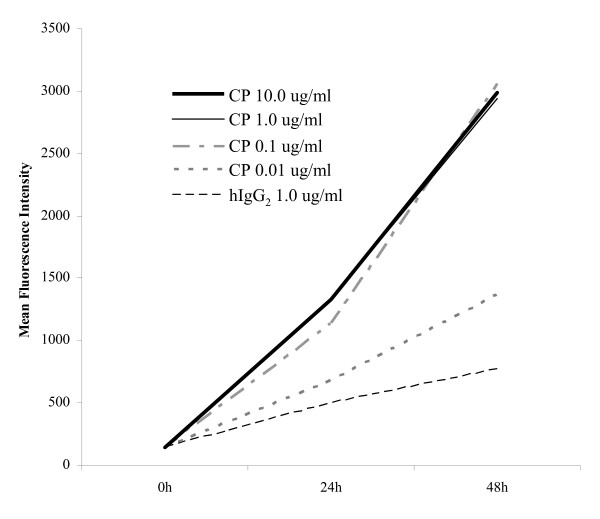
**B cell CD86 expression in response to titrated amounts of the CD40 mAb CP-870,893**. CD19+ B cells were purified from PBMC by negative selection and stimulated in the presence of hIgG2 or the indicated concentrations of CP-870,893 mAb. Cell surface CD86 expression was measured as mean fluorescence intensity pre-stimulation and at 24 hr and 48 hr after stimulation using flow cytometry. Results shown are for one donor and representative of three evaluated.

To measure the effects of CP-870,893 on human B cells, we first established the biologically optimal concentration to use in vitro. PBMC were enriched for CD19+ B cells and cultured in the presence of varying concentrations of either CP-870,893 or negative control hIgG2. Cells were analyzed by flow cytometry for viability and expression of cell surface molecules at baseline and at 24 and 48 hr subsequent to stimulation. A concentration of 1 ug/ml of CP-870,893 was sufficient to induce maximal expression of CD86 (Figure 1), as well as CD54, MHC class I, and MHC class II (data not shown). This concentration corresponds closely to the serum concentration of CP-870,893 previously reported for cancer patients 4-8 hr after receiving a single, intravenous infusion of the drug at the maximum tolerated dose of 0.2 mg/kg [14].

### Activation marker expression by in vitro stimulated B cells

To determine whether CP-870,893 activates B cells based on up-regulation of cell surface markers, purified total CD19+ B cells were incubated with either 1 ug/ml CP-870,893 or negative control hIgG2. After 48 hr, B cell expression of CD40, MHC Class I, MHC Class II, CD86, and CD70 were evaluated by flow cytometry. As shown in Table 1, expression of all markers was significantly increased for total B cells incubated with CP-870,893 as compared to the negative control hIgG2. Effects ranged from 2-fold (MHC class I) to more than 5-fold increases (MHC class II) over control. Given that CD19+ CD27+ memory and CD19+ CD27^neg ^naïve B cells respond differentially to maximum stimulatory signals [24], we also determined whether both these subsets could be activated by CP-870,893 alone. Similar to the effect for total CD19+ B cells, expression of all activation markers for both CD19+ CD27+ and CD19+ CD27^neg ^B cells was significantly increased after CP-870,893 stimulation compared to negative control (Table 1). Because TLR agonists synergize with CD40 stimulation in vivo in mice and in vitro for human DC [25,26], we evaluated the additive effects of the TLR9 ligand CpG ODN 2006 on CP-870,893-stimulated B cells. We first established that expression levels of all activation markers were significantly increased when total CD19+ B cells, CD19+ CD27+ memory B cells, or CD19+ CD27^neg ^naïve B cells were incubated for 48 hr in vitro with 1 ug/ml of CpG ODN 2006 as compared to ODN negative control (Table 1). For most markers, and in particular for CD40 and MHC class I, incubation with CpG ODN 2006 induced statistically significantly higher levels of surface marker expression than incubation with CP-870,893, a finding observed for total CD19+ B cells and each of the two CD27-defined subsets (Table 1). Dual incubation with CP-870,893 and CpG ODN 2006 compared to CP-870,893 alone led to significantly higher activation marker expression for all B cell subsets (Table 1), with the only exception being MHC Class II expression on CD19+ CD27^neg ^naïve B cells. In contrast, dual incubation with CP-870,893 and CpG ODN 2006 compared to CpG ODN 2006 alone induced higher expression of activation markers only for total B cells and CD27+ memory B cells and only for some, not all, markers. There was no statistical difference in surface marker upregulation for CD27^neg ^naïve B cells comparing CpG ODN 2006 plus CP-870,893 incubation to CpG ODN 2006 alone (Table 1). One-sided tests of interaction were not significant for any activation marker displayed in Table 1, thus we conclude that dual incubation does not yield more-than-additive effects. These results suggest that both memory and naïve B cells can be activated by the drug CP-870,893, and this CP-870,893 effect can be increased by the addition of CpG ODN 2006. Naïve B cells, as defined by lack of CD27 expression, appear relatively more responsive to CpG ODN 2006 than CP-870,893, and the addition of CP-870,893 to naïve B cells incubated with CpG ODN 2006 does not add significantly to upregulation of activation markers.

**Table 1:** B cell activation marker expression in response to stimulation

	**Negative control stimulation**		**CP-870,893 (CP)**		**CpG ODN 2006** **(CpG)**		**CP-870,893 plus CpG ODN 2006**		**Linear mixed effects model** p value*				
					
	**Mean**	**SE**	**Mean**	**SE**	**Mean**	**SE**	**Mean**	**SE**	**CP v. neg**	**CpG v. neg**	**CP v. CpG**	**CP+CpG v. CP**	**CP+CpG v. CpG**
					
**Total CD19+**^†^													
**CD40 MFI**	1928	92	3867	265	8828	738	10308	776	**<0.001**	**<0.001**	**<0.001**	**<0.001**	**0.004**
**MHC I MFI**	10623	591	23221	2098	27165	2026	40067	3481	**<0.001**	**<0.001**	**0.001**	**<0.001**	**<0.001**
**MHC II MFI**	30642	4979	82839	4675	83856	4703	108161	5250	**<0.001**	**<0.001**	0.91	**0.009**	**0.01**
**%CD86+**	15.7	3.3	58.6	5.3	73.8	4.4	82.6	3.4	**<0.001**	**<0.001**	0.05	**0.003**	0.34
**%CD70+**	7.7	2.6	33.5	4.8	39.2	6.1	51.2	5.9	**<0.001**	**<0.001**	0.25	**<0.001**	**0.01**
													
**CD19+ CD27+**^‡^													
**CD40 MFI**	2293	75	5625	524	13225	879	13951	680	**<0.001**	**<0.001**	**<0.001**	**<0.001**	0.56
**MHC I MFI**	15661	1650	30927	2008	37825	2687	53246	4910	**<0.001**	**<0.001**	**0.002**	**<0.001**	**<0.001**
**MHC II MFI**	33254	3509	98699	5195	97553	4251	121932	6400	**<0.001**	**<0.001**	0.89	**0.007**	**0.02**
**%CD86+**	27.3	2.7	64.5	5.0	74.0	3.5	83.5	2.5	**<0.001**	**<0.001**	0.10	**0.002**	0.17
**%CD70+**	18.8	1.7	56.2	1.9	62.3	2.1	74.4	2.6	**<0.001**	**<0.001**	**0.02**	**<0.001**	**0.001**
													
**CD19+ CD27negative**^‡^													
**CD40 MFI**	2195	108	4413	368	10417	1003	10622	804	**<0.001**	**<0.001**	**<0.001**	**<0.001**	0.88
**MHC I MFI**	7770	759	17726	1499	20993	2504	26777	4267	**<0.001**	**<0.001**	**0.010**	**<0.001**	0.14
**MHC II MFI**	40924	2749	90576	3333	86918	3847	96397	6585	**<0.001**	**<0.001**	0.60	0.47	0.22
**%CD86+**	13.2	1.6	61.1	5.1	75.8	4.9	82.8	3.5	**<0.001**	**<0.001**	**<0.001**	**<0.001**	0.13
**%CD70+**	5.0	0.6	30.3	3.3	34.8	3.8	41.3	5.1	**<0.001**	**<0.001**	**0.009**	**<0.001**	0.26

*Bold indicates p < 0.05

^†^n = 8 normal donors ^‡^n = 7 normal donors

### Cytokine secretion by in vitro stimulated B cells

To determine whether CP-870,893 induces human B cells to produce cytokines, supernatant from stimulated B cells was collected at 48 hr and analyzed for the presence of IL-6 and IL-10. IL-6 and IL-10 were studied because of their critical role in B cell physiology. IL-10 interrupts memory B cell formation [27], is a major plasma cell differentiation factor [28], and promotes in vitro differentiation of germinal center B cells into plasma cells [29]. IL-10 has also been shown to be a potent growth and differentiation factor for activated human B lymphocytes [30]. IL-6 is required for plasmablast differentiation and is an important plasma cell survival signal [31,32]. Activated B cells secrete IL-6 and IL-10, but there may be subsets of B cells with differential abilities to secrete cytokines [33].

**Figure 2:**
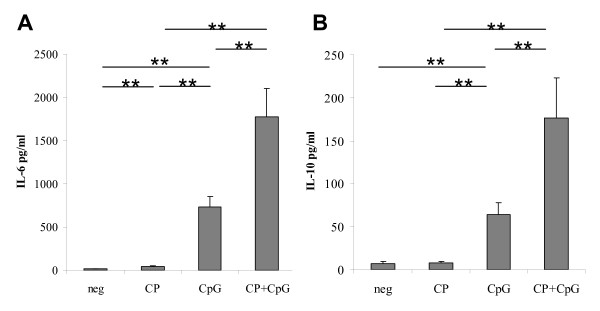
**CD19+ B cell cytokine secretion in response to in vitro stimulation**. Purified CD19+ B cells were stimulated with the negative control hIgG2 antibody and control ODN (neg), CD40 agonist mAb CP-870,893 (CP), CpG ODN 2006 (CpG), or both CP-870,893 and CpG ODN 2006 (CP + CpG). **(A) **IL-6 and **(B) **IL-10 concentrations were measured using cytokine bead array of supernatant at 48 hr. Mean values for 7 donors tested are shown with standard deviations. ** indicates p < 0.01 for the comparisons shown.

A trace amount of IL-6 (16.8 + 2.5 pg/ml) was measured in the supernatant of control stimulated total B cells, and this increased about four-fold (to 43.4 + 10.5 pg/ml, p < 0.05) in the supernatant of cells stimulated with CP-870,893. Small amounts of IL-10 were detected in the supernatant of B cells treated with CP-870,893 and control, with no statistical difference (Figure 2). In contrast, CpG ODN 2006 induced higher amounts of both IL-6 (731.5 + 122.7 pg/ml) and IL-10 (64.1 + 14.1) compared to CP-870,893 alone (Figure 2). Dual stimulation with CP-870,893 plus CpG ODN 2006 resulted in the highest levels of IL-6 (1779.9 + 327.4 pg/ml) and IL-10 (176.2 + 47.1 pg/ml), in each case significantly higher than cytokine production from stimulation with either reagent alone (Figure 2). Tests of interaction were not significant, demonstrating that dual incubation did not yield more-than-additive effects. Among the other cytokines tested in this assay (TNF-alpha, IL-1beta, and IL-12p70), cytokine production was undetectable in any of the experimental conditions. These results provide further evidence that TLR9 ligation can increase CP-870,893 activation of B cells.

### T cell stimulatory capacity of CP-870,893-activated B cells

**Figure 3:**
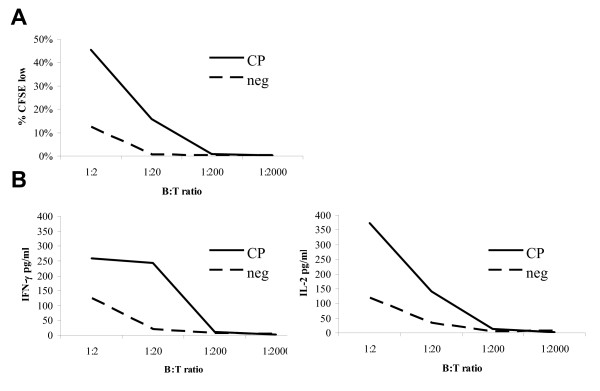
**Effect of CP-870,893 on T cell stimulatory capacity of B cells**. Purified CD19+ B cells from each of 7 donors were stimulated as described in Figure 2, irradiated, then co-cultured for 5 days with CFSE-labeled purified allogeneic CD4+ T cells at the indicated B cell:T cell titrations. **(A) **Percentage of CFSE^low ^T cells and **(B) **T cell IFN-gamma production (left panel) or T cell IL-2 production (right panel) for T cells incubated with CP-870,893-stimulated CD19+ B cells (solid line) or T cells incubated with negative control-stimulated CD19+ B cells (dashed line) at the indicated B cell to T cell ratios. Mean values for 7 donors tested at each condition are plotted and statistics for B:T ratio equal to 1:2 are given in the text. CP, CP-870,893 incubation; neg, negative control.

Because it is well-established that properly activated B cells can function as professional antigen presenting cells [21,34], we hypothesized that activation with CP-870,893 would enhance B cell capacity to stimulate T cells. To evaluate this, mixed lymphocyte reactions (MLR) were conducted in which B cells stimulated for 48 hr with either CP-870,893 or hIgG2 negative control were co-incubated with allogeneic CD4+ T cells. B cell stimulatory function was evaluated by measuring T cell proliferation and T cell cytokine secretion after 5 days of co-incubation. CP-870,893-activated B cells induced higher amounts of T cell proliferation than negative control B cells (e.g. 45.6% + 4.4% vs. 12.5% + 4.0% at a B cell to T cell ratio of 1:2, p < 0.001) (Figure 3A). Moreover, T cells stimulated with CP-870,893-activated B cells produced higher amounts of IFN-γ secretion than T cells stimulated with negative-control B cells (258.5 + 56.3 pg/ml vs. 122.7 + 37.6 pg/ml, at a B cell to T cell ratio of 1:2, p = 0.002) (Figure 3B). A similar pattern was observed for T cell IL-2 secretion (373.1 + 60.0 pg/ml vs. 118.5 + 32.4 pg/ml, at a B cell to T cell ratio of 1:2, p < 0.001) (Figure 3B). When purified CD19+ CD27+ memory B cells were used as stimulators in the MLR under the same conditions, CP-870,893-stimulated memory B cells were also able to induce significantly higher amounts of T cell proliferation (p < 0.001), IFN-γ (p < 0.001), and IL-2 (p < 0.001) secretion compared to negative control B cells (data not shown). For CD19+ CD27^neg ^naïve B cells, CP-870,893-stimulated B cells induced significantly higher proliferation (p < 0.001) and IL-2 (p = 0.004) compared to control B cells, but IFN-γ secretion was not significantly higher (p = 0.32) (data not shown). In summary, this data supports the hypothesis that CP-870,893 activation of B cells induces effective T cell stimulatory function, although less strongly for CD19+ CD27^neg ^naïve B cells.

### Dual stimulation of B cells via CD40 and TLR9 enhances B cell stimulatory capacity

**Figure 4:**
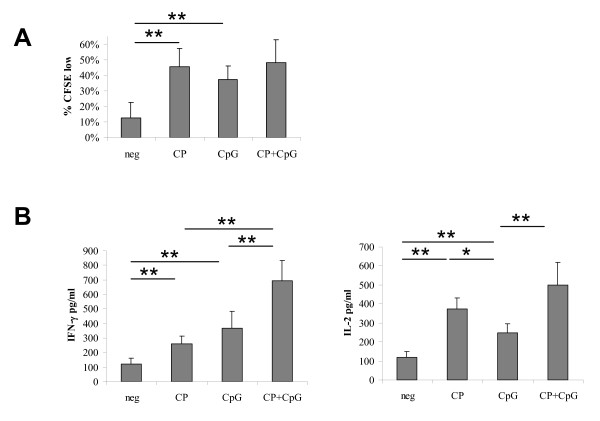
**CpG enhances CP-870,893-mediated T cell stimulatory capacity of B cells**. Purified CD19+ B cells were stimulated as in Figure 2 and used as stimulators in an MLR as described in Figure 3. **(A) **Percentage of CFSE^low ^T cells and **(B) **T cell IFN-gamma production (left panel) or T cell IL-2 production (right panel) are shown for responding T cells at a B cell to T cell ratio of 1:2. Mean values for 7 donors tested are shown with standard deviations. * indicates p < 0.05 for the comparisons shown, ** indicates p < 0.01. neg, negative control; CP, CP-870,893 incubation; CpG, CpG ODN 2006 incubation.

Since dual stimulation of B cells via TLR9 and CD40 resulted in increased activation as compared to single agent stimulation, we reasoned that the addition of CpG ODN 2006 stimulation to CP-870,893 might also augment T cell stimulatory capacity of activated B cells. CD19+ B cells were therefore stimulated with negative control reagents, CP-870,893 alone, CpG ODN 2006 alone, or CP-870,893 plus CpG ODN 2006 and used as stimulators in MLR. Although CpG-activated B cells induced significantly higher T cells proliferation (37.4% + 3.2%, p < 0.001) than negative control B cells, proliferation induced by dually stimulated B cells (48.1% + 5.6%) was not significantly higher than that induced by either CP-870,893-activated (p = 0.86) or CpG-activated (p = 0.26) B cells (Figure 4A). CpG-activated B cells also induced significantly higher T cell production IFN-γ (366.6 + 116.5 pg/ml, p = 0.001) and IL-2 (248.1 + 47.3 pg/ml, p < 0.001) compared to control B cells, but in this case, T cell IFN-γ secretion (692.7 + 138.8 pg/ml) in the MLR was significantly higher for dually stimulated B cells than for B cells stimulated with either CP-870,893 (p < 0.001) or CpG-activated (p = 0.002) alone (Figure 4B). Likewise, dually stimulated B cells induced a significantly higher amounts of T cell IL-2 (501.0 + 116.3 pg/ml) than CpG-activated B cells (p = 0.003), but this relationship was not significant for dually stimulated vs. CP-870,893-activated B cells (p = 0.33) (Figure 4B). Tests of interaction were not significant, demonstrating that dual incubation did not yield more-than-additive effects. Taken together, these results suggest that TLR9 agonists such as CpG ODN 2006 can increase the ability of CP-870,893 to induce T cell stimulatory capacity of B cells.

## Discussion

CD40 activation of APC plays an important role in driving anti-tumor T cell-mediated immune responses, and agonist CD40 antibodies which mimic the action of CD40 ligand are thought to represent promising therapeutics for novel immune strategies for cancer [9]. In this study, we evaluated the potential of the fully human agonist CD40 mAb CP-870,893 to activate human B cells and trigger T cell responses in vitro. CP-870,893 has been evaluated in phase I clinical trials for the treatment of advanced solid tumor malignancies and shown early signs of clinical efficacy, especially in patients with melanoma [14]. The primary pharmacodynamic effect of CP-870,893 has been a rapid decrease in circulating B cells associated with upregulation of CD86 expression on B cells that remain in circulation after infusion [14] (JR and RHV, unpublished observations). We now report direct evidence that CP-870,893 activates human B cells, including classically defined memory and naïve subsets, triggering increased expression of immuno-stimulatory molecules and production of cytokines. Furthermore, we found that CP-870,893-stimulated B cells induce proliferation of alloreactive T cells that secrete effector cytokines such as IFN-gamma and IL-2. These results underscore the agonistic effects of CP-870,893 and demonstrate that the antibody can accomplish an activation state of resting human B cells consistent with licensed APC. Clinically, for patients receiving CP-870,893, there may be a link between the ability of CP-870,893 to activate B cells and the rapid (but transient) depletion of CD19+ B cells from circulation after infusion if cell adhesion molecules and chemokine receptors as also upregulated in vivo as part of activation. In vitro, we have observed increases in CD54 and CCR7 (10-fold and 1.4-fold increase in MFI, respectively) following 48 hr incubation of purified B cells with CP-870,893 (data not shown), which supports a hypothesis that CP-870,893 activation might drive circulating B cells into tumor, lymph nodes, or spleen. It should be noted, however, that acute splenomegaly or lymph node swelling has not be observed in patients following CP-870,893 infusion [14].

By further evaluating CP-870,893 in combination with CpG ODN 2006, we also found in this study that TLR9 signalling augments the action of CP-870,893 on B cell marker expression, B cell cytokine production, and alloreactive T cell IFN-gamma production for both memory and naïve B cell subsets. Clinical grade versions of CpG ODN 2006 have already undergone clinical testing [35-39], and one formulation, PF-3512676, is owned by the same manufacturer as CP-870,893, which heightens the translational potential of combining CD40 and TLR9 stimulation in patients. Although the mechanism of the augmented effect with dual stimulation remains to be fully explained, the signalling pathways of CD40 and TLR9 are largely distinct from each other proximally but distally share some common signalling nodes such as NFkappaB and MAP kinases [9]. Moreover, in mice, positive effects of dual CD40 and TLR activation have been well-described [13,26], providing further pre-clinical rationale to test CD40/TLR9 combined therapy in human cancer patients. Expansion of antigen-specific T cells, for example, is enhanced with the use of CD40 and TLR agonists [26]. A more recent analysis of combined vs. monotherapy in a mouse melanoma model showed that combined activation via CD40 and TLR9 results in tumor-infiltrating CD8+ T cells at a very high frequency and with potent anti-tumor activity [13]. Because, however, TLR9 expression significantly differs between mice and humans, mouse studies are not fully relevant to human translational efforts in this regard [38], and the current work is needed to demonstrate the physiological impact of clinical grade CD40 agonists in patients.

Our data provides evidence that combined CD40 and TLR9 signalling, and in particular CP-870,893 plus CpG ODN 2006, induces activation of human B cells more than either agent alone. Taken together, these findings suggest that the combination of CP-870,893 and CpG ODN 2006 represents a practical - and available - clinical approach to test the hypothesis that dual CD40/TLR9 activation in vivo can promote tumor immunity in patients.

We have recently reported that patients with advanced solid tumors exhibit marked disturbances in B cell homeostasis, manifest in particular by a collapse of the circulating CD27+ memory B cell population [24]. We therefore studied both CD27+ memory B cells and CD27^neg ^naïve B cells in this investigation. We found that CP-870,893 was effective at activating either subset, but as expected, CD27^neg ^B cells appeared relatively hyporesponsive to CP-870,893 compared to CD27+ B cells. CD27^neg ^B cells also appeared relatively hyporesponsive to stimulation with CpG ODN 2006 or combined CP-870,893 and CpG ODN 2006 stimulation. For CD27^neg ^B cells but not CD27+ memory B cells, the addition of CP-870,893 did not increase the activation achieved with CpG ODN 2006 alone (whereas the addition of CpG ODN 2006 did increase activation from CP-870,893 alone). Although our results do not suggest that CP-870,893 and CpG ODN 2006 are synergistic, these results do suggest that the inclusion of TLR9 stimulation is important for optimal activation of naïve B cells, a finding of particular importance for patients with advanced cancer in whom naïve B cells dominate the peripheral B cell compartment [24]. Indeed, TLR stimulation may be a universal requirement for the full elaboration of any human B cell function, as it has been recently shown that that TLR stimulation simultaneously with ligation of CD40 and the B cell antigen receptor is required for full activation of naive human B cells and production of antibodies in T-dependent immune responses [40].

To what extent does CP-870,893-mediated B cell activation matter therapeutically, particularly if it has already been established that CP-870,893 activates DC [22]? Although measurements of B cell modulation following infusion of CP-870,893 were initially pursued purely as a potential pharmacodynamic measurement following drug delivery, we hypothesize that B cell activation might directly contribute at least in part to the mechanisms of action of the antibody. It has become increasingly appreciated that resting B cells regulate peripheral immune tolerance. As shown in multiple murine models, elimination of peripheral B cells increases the potency of cancer vaccination and improves cellular immunity [15-19]. In humans, the use of CD20 mAb rituximab to eliminate peripheral B cells in patients undergoing renal allograft transplantation results in acute (T cell mediated) graft rejection in 83% of subjects despite ongoing systemic immunosuppression [41], findings that dramatically underscore the critical role resting B cells can play in mediating immune T cell tolerance. In light of classic studies that tolerogenic B cells in mice can be converted to stimulatory cells following CD40-mediated activation [21], our findings raise the hypothesis that CP-870,893 acting as a potent and selective agonist of CD40 may have a similar pro-immunity effect on B cells in humans. Establishing evidence to support this hypothesis becomes an important goal of future clinical trials with CP-870,893.

In summary, our findings provide several important areas of insight with regard to CP-870,893 as an anti-cancer immune therapy. First, CP-870,893 induces activation of highly purified B cells that were isolated without manipulation from peripheral blood and evaluated in short-term assays, demonstrating that the mAb is agonistic. Second, CP-870,893-activated B cells are able to trigger proliferation of T cells that secrete high levels of effector cytokines, suggesting a potential role for CP-873,893 in licensing CD40-expressing APC in humans to enable high quality T cell responses. Third, the effects of CP-870,893 on B cells can be increased with simultaneous TLR9 stimulation. If as suggested by elegant mechanistic studies in mouse models [2-7], the therapeutic goal of CD40 agonists is to activate APC to trigger T cell immunity in patients, our data and that of others [13,26,42] provide a rationale for clinical strategies that combine CD40 activation with TLR9 ligation.

## Conclusion

Our data demonstrate that the clinical CD40 mAb CP-870,893 is agonistic and activates naïve and memory B cells with properties consistent with licensed APC. B cell activation with CP-870,893 can be further increased with TLR9 co-stimulation and can be accomplished with available clinical grade reagents.

## Competing interests

RHV receives clinical and laboratory research funding from Pfizer Corp. The authors declare that they have no other competing interests.

## Authors' contributions

The studies were designed by ELC, JR, and RHV and were performed by ELC. ELC and RHV wrote the paper together with RM and JR. RM provided all statistical analyses. All authors read and approved the final manuscript.

## References

[B1] Lanzavecchia A (1998). Immunology. Licence to kill. Nature.

[B2] Bennett SR, Carbone FR, Karamalis F, Flavell RA, Miller JF, Heath WR (1998). Help for cytotoxic-T-cell responses is mediated by CD40 signalling. Nature.

[B3] Ridge JP, Di Rosa F, Matzinger P (1998). A conditioned dendritic cell can be a temporal bridge between a CD4+ T- helper and a T-killer cell. Nature.

[B4] Schoenberger SP, Toes RE, Voort EI van der, Offringa R, Melief CJ (1998). T-cell help for cytotoxic T lymphocytes is mediated by CD40-CD40L interactions. Nature.

[B5] French RR, Chan HT, Tutt AL, Glennie MJ (1999). CD40 antibody evokes a cytotoxic T-cell response that eradicates lymphoma and bypasses T-cell help. Nat Med.

[B6] Diehl L, den Boer AT, Schoenberger SP, Voort EI van der, Schumacher TN, Melief CJ, Offringa R, Toes RE (1999). CD40 activation in vivo overcomes peptide-induced peripheral cytotoxic T-lymphocyte tolerance and augments anti-tumor vaccine efficacy. Nat Med.

[B7] Sotomayor EM, Borrello I, Tubb E, Rattis FM, Bien H, Lu Z, Fein S, Schoenberger S, Levitsky HI (1999). Conversion of tumor-specific CD4+ T-cell tolerance to T-cell priming through in vivo ligation of CD40. Nat Med.

[B8] van Mierlo GJ, den Boer AT, Medema JP, Voort EI van der, Fransen MF, Offringa R, Melief CJ, Toes RE (2002). CD40 stimulation leads to effective therapy of CD40(-) tumors through induction of strong systemic cytotoxic T lymphocyte immunity. Proc Natl Acad Sci USA.

[B9] Vonderheide RH (2007). Prospect of targeting the CD40 pathway for cancer therapy. Clin Cancer Res.

[B10] Armitage RJ, Fanslow WC, Strockbine L, Sato TA, Clifford KN, Macduff BM, Anderson DM, Gimpel SD, Davis-Smith T, Maliszewski CR (1992). Molecular and biological characterization of a murine ligand for CD40. Nature.

[B11] van Kooten C, Banchereau J (2000). CD40-CD40 ligand. J Leukoc Biol.

[B12] Quezada SA, Jarvinen LZ, Lind EF, Noelle RJ (2004). CD40/CD154 interactions at the interface of tolerance and immunity. AnnuRevImmunol.

[B13] Ahonen CL, Wasiuk A, Fuse S, Turk MJ, Ernstoff MS, Suriawinata AA, Gorham JD, Kedl RM, Usherwood EJ, Noelle RJ (2008). Enhanced efficacy and reduced toxicity of multifactorial adjuvants compared with unitary adjuvants as cancer vaccines. Blood.

[B14] Vonderheide RH, Flaherty KT, Khalil M, Stumacher MS, Bajor DL, Hutnick NA, Sullivan P, Mahany JJ, Gallagher M, Kramer A, Green SJ, O'Dwyer PJ, Running KL, Huhn RD, Antonia SJ (2007). Clinical activity and immune modulation in cancer patients treated with CP-870,893, a novel CD40 agonist monoclonal antibody. J Clin Oncol.

[B15] Qin Z, Richter G, Schuler T, Ibe S, Cao X, Blankenstein T (1998). B cells inhibit induction of T cell-dependent tumor immunity. Nat Med.

[B16] Perricone MA, Smith KA, Claussen KA, Plog MS, Hempel DM, Roberts BL, St George JA, Kaplan JM (2004). Enhanced efficacy of melanoma vaccines in the absence of B lymphocytes. J Immunother.

[B17] Shah S, Divekar AA, Hilchey SP, Cho HM, Newman CL, Shin SU, Nechustan H, Challita-Eid PM, Segal BM, Yi KH, Rosenblatt JD (2005). Increased rejection of primary tumors in mice lacking B cells: inhibition of anti-tumor CTL and TH1 cytokine responses by B cells. Int J Cancer.

[B18] Inoue S, Leitner WW, Golding B, Scott D (2006). Inhibitory effects of B cells on antitumor immunity. Cancer Res.

[B19] Kim S, Fridlender ZG, Dunn R, Kehry MR, Kapoor V, Blouin A, Kaiser LR, Albelda SM (2008). B-cell depletion using an anti-CD20 antibody augments antitumor immune responses and immunotherapy in nonhematopoetic murine tumor models. J Immunother.

[B20] Bouaziz JD, Yanaba K, Tedder TF (2008). Regulatory B cells as inhibitors of immune responses and inflammation. Immunol Rev.

[B21] Buhlmann JE, Foy TM, Aruffo A, Crassi KM, Ledbetter JA, Green WR, Xu JC, Shultz LD, Roopesian D, Flavell RA (1995). In the absence of a CD40 signal, B cells are tolerogenic. Immunity.

[B22] Hunter TB, Alsarraj M, Gladue RP, Bedian V, Antonia SJ (2007). An agonist antibody specific for CD40 induces dendritic cell maturation and promotes autologous anti-tumour T-cell responses in an in vitro mixed autologous tumour cell/lymph node cell model. Scand J Immunol.

[B23] Diggle PJ, Liang KY, Zeger SL (1994). Analysis of longitudinal data.

[B24] Carpenter EL, Mick R, Rech AJ, Beatty GL, Colligon TA, Rosenfeld MR, Kaplan DE, Chang KM, Domchek SM, Kanetsky PA, Fecher LA, Flaherty KT, Schuchter LM, Vonderheide RH (2009). Collapse of the CD27+ B-cell compartment associated with systemic plasmacytosis in patients with advanced melanoma and other cancers. Clin Cancer Res.

[B25] Krug A, Towarowski A, Britsch S, Rothenfusser S, Hornung V, Bals R, Giese T, Engelmann H, Endres S, Krieg AM, Hartmann G (2001). Toll-like receptor expression reveals CpG DNA as a unique microbial stimulus for plasmacytoid dendritic cells which synergizes with CD40 ligand to induce high amounts of IL-12. Eur J Immunol.

[B26] Ahonen CL, Doxsee CL, McGurran SM, Riter TR, Wade WF, Barth RJ, Vasilakos JP, Noelle RJ, Kedl RM (2004). Combined TLR and CD40 triggering induces potent CD8+ T cell expansion with variable dependence on type I IFN. J Exp Med.

[B27] Calame KL (2001). Plasma cells: finding new light at the end of B cell development. Nat Immunol.

[B28] Arce E, Jackson DG, Gill MA, Bennett LB, Banchereau J, Pascual V (2001). Increased frequency of pre-germinal center B cells and plasma cell precursors in the blood of children with systemic lupus erythematosus. J Immunol.

[B29] Choe J, Choi YS (1998). IL-10 interrupts memory B cell expansion in the germinal center by inducing differentiation into plasma cells. Eur J Immunol.

[B30] Rousset F, Garcia E, Defrance T, Peronne C, Vezzio N, Hsu DH, Kastelein R, Moore KW, Banchereau J (1992). Interleukin 10 is a potent growth and differentiation factor for activated human B lymphocytes. Proc Natl Acad Sci USA.

[B31] Kawano MM, Mihara K, Huang N, Tsujimoto T, Kuramoto A (1995). Differentiation of early plasma cells on bone marrow stromal cells requires interleukin-6 for escaping from apoptosis. Blood.

[B32] Grammer AC, Lipsky PE (2002). CD154-CD40 interactions mediate differentiation to plasma cells in healthy individuals and persons with systemic lupus erythematosus. Arthritis Rheum.

[B33] Harris DP, Haynes L, Sayles PC, Duso DK, Eaton SM, Lepak NM, Johnson LL, Swain SL, Lund FE (2000). Reciprocal regulation of polarized cytokine production by effector B and T cells. Nat Immunol.

[B34] Fuchs EJ, Matzinger P (1992). B cells turn off virgin but not memory T cells. Science.

[B35] Cooper CL, Davis HL, Morris ML, Efler SM, Adhami MA, Krieg AM, Cameron DW, Heathcote J (2004). CPG 7909, an immunostimulatory TLR9 agonist oligodeoxynucleotide, as adjuvant to Engerix-B HBV vaccine in healthy adults: a double-blind phase I/II study. J Clin Immunol.

[B36] Speiser DE, Lienard D, Rufer N, Rubio-Godoy V, Rimoldi D, Lejeune F, Krieg AM, Cerottini JC, Romero P (2005). Rapid and strong human CD8+ T cell responses to vaccination with peptide, IFA, and CpG oligodeoxynucleotide 7909. J Clin Invest.

[B37] Molenkamp BG, van Leeuwen PA, Meijer S, Sluijter BJ, Wijnands PG, Baars A, Eertwegh AJ van den, Scheper RJ, de Gruijl TD (2007). Intradermal CpG-B activates both plasmacytoid and myeloid dendritic cells in the sentinel lymph node of melanoma patients. Clin Cancer Res.

[B38] Krieg AM (2007). Development of TLR9 agonists for cancer therapy. J Clin Invest.

[B39] Molenkamp BG, Sluijter BJ, van Leeuwen PA, Santegoets SJ, Meijer S, Wijnands PG, Haanen JB, Eertwegh AJ van den, Scheper RJ, de Gruijl TD (2008). Local administration of PF-3512676 CpG-B instigates tumor-specific CD8+ T-cell reactivity in melanoma patients. Clin Cancer Res.

[B40] Ruprecht CR, Lanzavecchia A (2006). Toll-like receptor stimulation as a third signal required for activation of human naive B cells. Eur J Immunol.

[B41] Clatworthy MR, Watson CJ, Plotnek G, Bardsley V, Chaudhry AN, Bradley JA, Smith KG (2009). B-cell-depleting induction therapy and acute cellular rejection. N Engl J Med.

[B42] Wagner M, Poeck H, Jahrsdoerfer B, Rothenfusser S, Prell D, Bohle B, Tuma E, Giese T, Ellwart JW, Endres S, Hartmann G (2004). IL-12p70-dependent Th1 induction by human B cells requires combined activation with CD40 ligand and CpG DNA. J Immunol.

